# Supplementary material for: Urine Metabolome Dynamics Discriminate Influenza Vaccination Response
Source: Viruses. 2023 Jan 14;15(1):242. doi: 10.3390/v15010242 (PMC9861122; doi:10.3390/v15010242)
Supplement: Supplementary file 1 [file viruses-15-00242-s001.zip › Supplementary Figures S1 and S2.pdf]

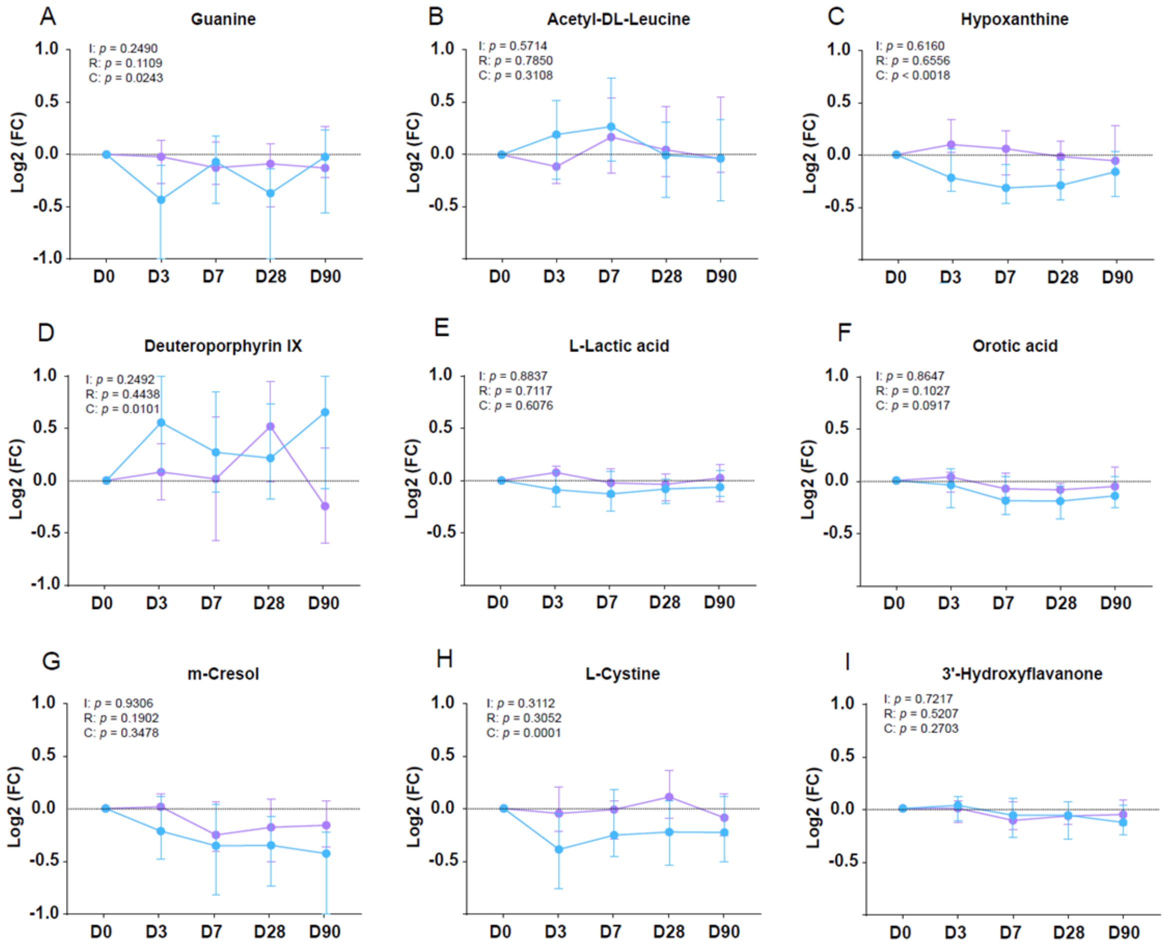

Supplementary Figure S1. Metabolite time-course dynamics of IAV vaccine response (Adults Only). (**A-I**) The subset of adults <65 yr old were used for all panels. Line plot for the relative change in subject urine metabolite levels with respect to baseline over the 90-day time-course. For each metabolite, error bars represent standard deviation of fold-change compared to baseline, Y-axis represents Log2 values. Each metabolite was analyzed with a two-way ANOVA. I = Interaction of the two factors, R = Row factor, days post-vaccination, C = Column factor, Seroconversion status (High-responder or None-responder).

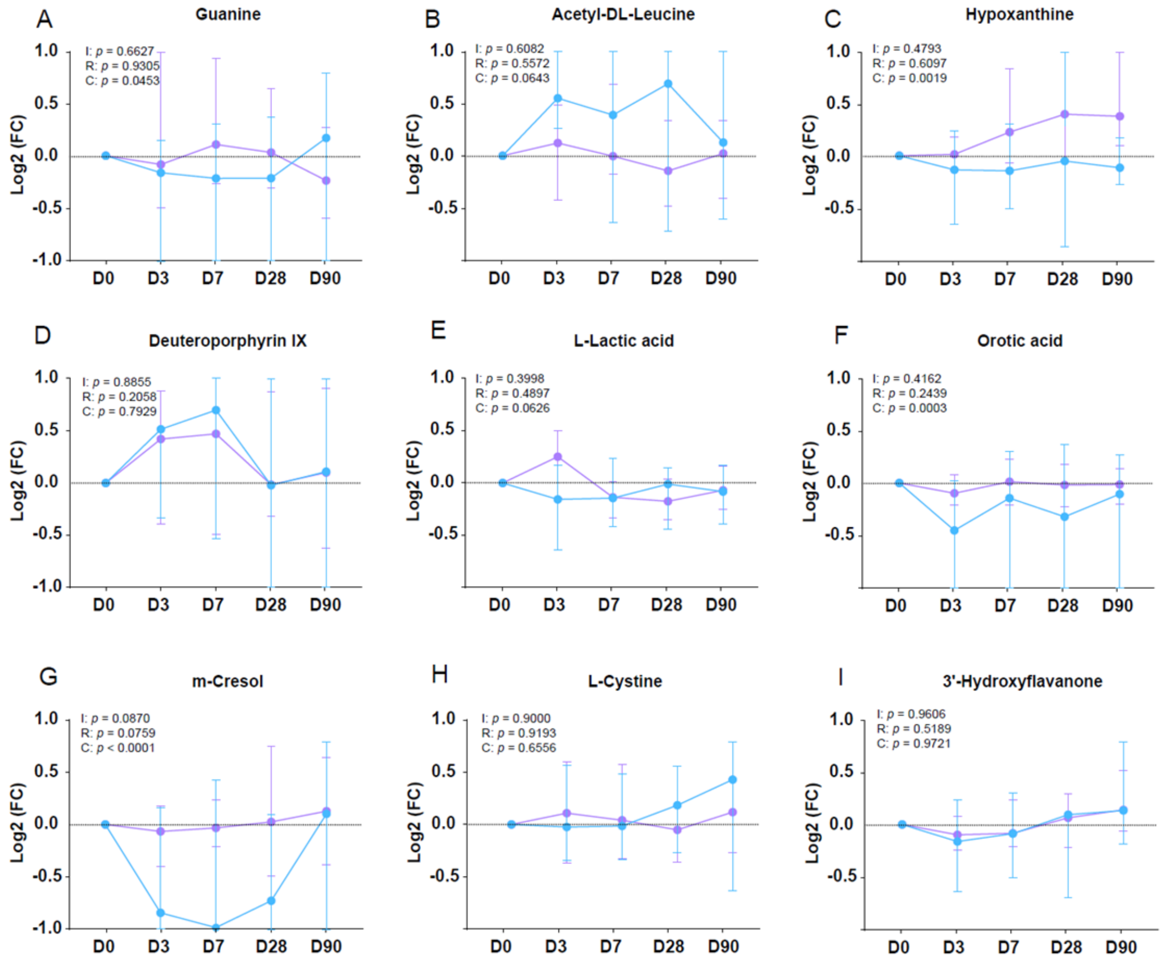

Supplementary Figure S2. Metabolite time-course dynamics of IAV vaccine response (Geriatric Only). (**A-I**) The subset of geriatric subjects ⩾ 65 yr old were used for all panels. Line plot for the relative change in subject urine metabolite levels with respect to baseline over the 90-day time-course. For each metabolite, error bars represent standard deviation of fold-change compared to baseline, Y-axis represents Log2 values. Each metabolite was analyzed with a two-way ANOVA. I = Interaction of the two factors, R = Row factor, days post-vaccination, C = Column factor, Seroconversion status (High-responder or None-responder).
